# That’s A Scientist Should Do—A Dialog with Tomas Lindahl

**DOI:** 10.1016/j.gpb.2019.07.001

**Published:** 2019-07-16

**Authors:** Shanshan Xie, Yuxia Jiao

**Affiliations:** Beijing Institute of Genomics, Chinese Academy of Sciences, Beijing 100101, China

Dr Tomas Lindahl is a world-renowned scientist specialized in cancer research, in particular, DNA repair [1]. In 2015, he was awarded the Nobel Prize in Chemistry jointly with Dr Paul L. Modrich and Dr Aziz Sancar “*for mechanistic studies of DNA repair*” (https://www.nobelprize.org/prizes/chemistry/2015/press-release/) [2]. During his recent visits in China, besides delivering lectures, Tomas also attended a couple of group meetings with students and PIs. He actively interacted with the audience, not only on topics relating to DNA repair, but also on how to do science and beyond. We presented this special report based on recordings.

## Amazing world of DNA repair

*Q1: Why did you choose to study DNA repair 40 years ago?*

Tomas: Basically I was interested in the instability of DNA and I found that DNA was unstable. This was unexpected at that time. One has to think of some strategies to counteract this instability, you cannot change the chemistry. We have to predict that there has to be an important role of DNA repair, to secure the stability of the DNA.

*Q2: What’s the difference between mutagenesis and error-prone DNA replication?*

Tomas: Well. It’s a good question because we don’t quite know what proportion of the DNA instability is due to direct errors of the replication machinery. The most likely answer is that the replication machinery is extremely accurate because it has had many millions of years to develop accuracy that is essential here. And, we know that accuracy is a key factor in replication of DNA, and that actually brings it together with the beginning of my talk, where I said that DNA is unstable, like deamination of cytosine to uracil in DNA. Why hasn't something better evolved? Well, organic chemists now can evolve more stable components that you can use to synthesize DNA. So why hasn’t nature chosen those instead? And the answer, in all cases that we looked at, is that they are more difficult to copy at very high level of accuracy. So, nature has found best compromise: you need to have a very high fidelity of replication and that restricts what kind of nucleotides you can have in the DNA. The DNA we have already is probably the best alternative. Then exactly how much of the damage of the DNA, that goes endogenously, is due to errors in replication machinery, and how much is due to instability of DNA? I think both are important and depends on the conditions. I think that probably the instability of DNA is the major factor, because the replication machinery has had plenty of time to evolve to become extremely accurate.

*Q3: Does the transcription machinery have the same or lower accuracy compared to the DNA replication machinery? Are there key factors in the replication complex to keep fidelity (high) or does the fidelity depend on the different polymerases used?*

Tomas: The question is whether DNA transcription is as accurate as DNA replication. Probably not. It’s very accurate of course, but it doesn’t have the same demands. If you make occasional mistake in transcribing DNA, it may not have any great effect because that particular RNA molecule is not active, and if it is, it doesn’t live very long. It is not problematic if the sequence of DNA is unchanged. DNA polymerases are not the same as RNA polymerases and DNA polymerases have been developed into accuracy.

*Q4: We need DNA damage repair system to repair damages in the DNA. Yet, if the system is too strong and repairs any error in the DNA, we will have no materials for evolution. What is your opinion on the relationship between DNA repair and evolution?*

Tomas: I think it is an important question. You are quite right if there were no changes in the DNA at all, we just wouldn’t evolve. There are so many things that cause changes in DNA structure, and I think the cell is just struggling keeping the mutation rate as low as possible due to endogenous damage. To reduce the mutation rate to zero is very difficult and the enzymes we have now are not close to that. So far, the problem has been how you can repair all the DNA damages you get exposed to, and I think much of them are endogenous damages from exposure to radiation or chemicals. So because of that, the cell will struggle to keep up all the time and to maintain a low mutation rate. One exception that I brought up is the antibody genes where it is useful and required to have active mutagenesis. We cannot have that kind of mutation-prone process in all our genes. But it’s a good and difficult question.

*Q5: Could the rate of DNA mutation be regulated or not? Is it under the pressure of natural selection?*

Tomas: The environment we are living in generates too much mutagenesis and all species struggle to keep the mutation rate down. From that point of view, I don’t think there is a need of an active mutation mechanism to speed up evolution. I may be wrong. I think you obviously need some changes on DNA otherwise there will be no evolution. And, if there are many changes on DNA, it will soon come to a point that there are too many mutations, so you need something in between to balance this. This might well be regulated. If there was some way we could avoid mutagenesis and use that further down that would be better. But, we need a little bit of mutation for evolution.

*Q6: The majority of the proteins and pathways in DNA replication has been identified from yeast. Is the DNA replication field still an open field, with any chance for new scientists to have achievements? In another way, since you, Dr Modrich, and Dr Sancar won the Nobel Prize for the DNA repair, is there any chance for next Nobel Prize to DNA replication?*

Tomas: As we all know that the prize committee try to answer if the prize goes to the same area over and over again. But, there had already been Nobel Prize for DNA replication. Arthur Kornberg discovered DNA polymerase which made us understand how DNA is replicated. This was a very important discovery and a beautiful piece of biochemistry. Then, there had been contributions in the fields of DNA replication and DNA recombination that have been recognized. These areas will come back in new course, not these years but some other years perhaps. Science progresses all the time and there will be more important discoveries that help understand DNA formation or other important issues.

*Q7: Could we apply the DNA repair mechanisms clinically, to repair the DNA or to avoid the disaster of DNA damage?*

Tomas: Up to couples of years ago, it sounded extremely difficult to be able to go into cells and correct specific base damage that turns out to be mutagenic and perhaps cancer-causing without affecting other parts of DNA. But, just over the last couples of years, there are new methods of genetic engineering that largely can answer that challenge. This is a fast-moving field and I can see that, based on recent publications, we should now be able to correct some inherited human diseases using genetic engineering. Some people are very afraid of genetic engineering in human cells, perhaps for good reasons, because they don’t want to have permanent changes in DNA, so this would have to be highly regulated. But some family is unfortunate enough to have a child with inherited well-defined mutation in the DNA. If we have the technology to correct that mutation, in that family, I am sure it should be allowed to do that. I can see there is a whole new field of regulation coming up here, because you don’t want parents coming up and saying, “We want to produce, by genetic engineering, a child with big muscles, so he can become a famous sportsman”.

*Q8: People believe that the radiation from mobile phone and computer could cause DNA damage; particularly pregnant women should reduce the use of these devices because they think it might lead to having an unhealthy baby. Is it a reasonable concern, or just an overworry?*

Tomas: There is a simple answer to that question. Radiation from a mobile phone is not so strong that it could break any covalent bonds. The key fact is that you can’t get mutation without altering the structure of the DNA. So, although there has been a lot of writing about radiation from mobile phone that could be damaging, I think there is zero risk of getting DNA damage by using mobile phone.

## Challenges and fun in doing science

*Q9: As a scientist, should we pursue the things which we are really interested in, or work on something in the hot fields that is easy to publish and to get more attention and funding to support our research?*

Tomas: These are so called ethical and organizational problems, which are important enough. What I talked about is biochemistry and understanding mutations, the cause of cancer, and what it leads to, primarily more knowledge. There are lots of things that we don’t understand and that need to be clarified. I think that instead of looking at science as a black box, we make our own contribution for help, make that box a little less black, and contribute some new knowledge to science, that’s going to be a good thing. I think there is no reason for not trying to understand the world you are living in. That is what a scientist should do, trying to contribute knowledge.

*Q10: Do you think hard work is an essential factor for doing science?*

Tomas: Yes. At times, hard work is very important. Especially when you have interesting observation and another laboratory may have similar observations. Then you have to work hard. It’s a competitive world of science and in situation like this everyone is working hard. Even more important than working hard is to think very carefully about what you are doing, and doing it in the most effective way. You don’t have to work so hard, if you come up with an easier or better way for doing it. Therefore when you work hard, you also need to think hard. If you work hard, it’s very important to select a really good project to work on. Because you can’t work hard on several different projects. You just have to focus. If you don’t like your project, go back to your supervisor and have open discussion about it or do it in a different way. You don’t want to waste your own time. You want to do something very interesting, targeted, and successfully. It is up to you to decide that you are doing it in that way.

*Q11: How do you arrange your time to balance work and life?*

Tomas: Well, science can be a hard master. You have to find enough time to do science. But everybody’s conditions are different. What suits one person doesn’t suit another person. It is important that you also protect your own life and are happy with what you are doing. You don’t want to work so hard that it becomes overbearing or that you feel under so much pressure that you don’t enjoy it. Because it’s important in science to enjoy what you are doing; you will burn out after a while if you do science and you don’t enjoy it, then you should probably change your tactics. You should only do what you enjoy doing. Science is very interesting. You know if you really like this job; that provides some of the motivation. You really want to know. If you have new interesting results and you are ahead of others with this, that’s a very exciting position to be in. What you are doing, nobody else has ever seen it before. That’s exciting.

*Q12: Experiments do not always go smoothly. How do you adjust your mood when you get negative results?*

Tomas: It’s a good question. You just have to realize that nature is sometimes smarter than you are. You have some idea and you spend some time on it. It turns out that that’s probably not the way things are working, because nature has designed some other mechanisms. In philosophy, you can come up with all kinds of different answers and constructs, argue about that. The nice thing with natural science is that there is the truth. So if you have two models for something, likely thing is that one model is right and the other is wrong, it doesn’t matter if you argue about which is your favourite model, because nature has already decided on it. You have to perform experiments to find out. Then if you spend some time on experiments that don’t work, at some points, you have to stop and say this is probably not the way it is working, think what is the alternative and work on that instead. I don’t know any scientist who is smart enough to always predict the right model to work on. With deeper knowledge you will be able to come up with different and better models to study and this could save you lots of time. But that is easy to say, when you are doing experiments it is very difficult to know what the right answer is going to be. You just have to do experiments. That’s why you have to work hard to do science. Never stop, never give up, you will find the answer at last.

## Competing interests

Both SX and YJ are editors of the journal.
